# Studying and Incorporating Efficiency into Gastrointestinal Endoscopy Centers

**DOI:** 10.1155/2015/764153

**Published:** 2015-05-25

**Authors:** Lukejohn W. Day, David Belson

**Affiliations:** ^1^Division of Gastroenterology, San Francisco General Hospital and Trauma Center, San Francisco, CA 94110, USA; ^2^Daniel J. Epstein Department of Industrial and Systems Engineering, University of Southern California, Los Angeles, CA 90089, USA

## Abstract

Efficiency is defined as the use of resources in such a way as to maximize the production of goods and services. Improving efficiency has been the focus of management in many industries; however, it has not been until recently that incorporating efficiency models into healthcare has occurred. In particular, the study and development of improvement projects aimed at enhancing efficiency in GI have been growing rapidly in recent years. This focus on improving efficiency in GI has been spurred by the dramatic rise in the demand for endoscopic procedures as well as the rising number of insured patients requiring GI care coupled at the same time with limited resources in terms of staffing and space in endoscopy centers. This paper will critically review the history of efficiency in endoscopy centers, first by looking at other healthcare industries that have extensively studied and improved efficiency in their fields, examine a number of proposed efficiency metrics and benchmarks in endoscopy centers, and finally discuss opportunities where endoscopy centers could improve their efficiency.

## 1. Introduction

Efficiency has many varying definitions and historically has been employed in economic models and businesses. At its core efficiency is defined as the use of resources in such a way as to maximize the production of goods and services [1]. While the study of efficiency has been the focus of management in many industries, it has not been until recently that incorporating efficiency models into healthcare has occurred. In particular, one area of healthcare where the study of efficiency has seen a particular increase is the endoscopy center in gastroenterology (GI) departments.

In the last several years there has been a dramatic surge in the request for GI specialty care and in particular endoscopic services [2–4]. Such a surge is expected to continue as the number of insured patients in the U.S. increases as a result of the Affordable Health Care Act. Consequently, hospitals and endoscopy centers have been challenged to provide quality GI healthcare with limited resources and a large, expanding patient population. It is under this scenario where the improvement of efficiency within the endoscopy center has numerous advantages and can help to tackle many of these challenges. This paper will critically review the history of efficiency in endoscopy centers, first by looking at other healthcare industries that have extensively studied and improved efficiency in their fields, examine a number of proposed efficiency metrics and benchmarks in endoscopy centers, and finally discuss opportunities where endoscopy centers could improve their efficiency.

## 2. Looking to Healthcare Counterparts to Begin the Process

In the field of healthcare efficiency, anesthesia has been the clear leader for the last four decades. Specifically, there has been a tremendous amount of research which is focused on improving efficiency in ambulatory surgery centers and hospital operating rooms (OR) and is an area from which the GI field can learn. From this expansive field of literature, three key steps emerge on how to improve efficiency within a healthcare setting. The first step is the establishment and use of metrics so that improvement work can be measured and monitored. Clearly defined metrics have been established by the Association of Anesthesia Clinical Directors which have been utilized and validated in several studies [5]. The second step is to better understand areas where performance improvement work is needed. In this arena anesthesia has been at the forefront of improving efficiency in healthcare through the reengineering of operations and processes in the operating room with notable improvements in patient care and quality [6–10]. Pivotal to this success is the employment of time and motion studies (e.g., direct and continuous observation of a task) in conjunction with discrete event simulation modeling (e.g., modeling the operations of a system). A number of healthcare specialties have employed simulation modeling to improve efficiency, such as primary care [11–14], emergency rooms [15–17], and pediatrics [18, 19], but anesthesia [20, 21] has been a leader in this area. For example, anesthesia simulation modeling has demonstrated that duration of a surgical procedure is not a rate limiting step in OR efficiency, but rather factors that occur prior to and subsequently after the procedure are more critical factors to OR efficiency [6]. The third and final step is the implementation of performance improvement projects using a systematic and consistent process, in most cases modeled after the PDSA (plan-do-study-act) process. On this topic, two important elements have emerged from the anesthesia literature with regard to implementing improvement projects: (1) clearly studying and identifying process inefficiencies and instituting multidisciplinary education programs with clear goals to address them [9, 10] and (2) use of parallel processing of tasks among staff members which can lead to a dramatic reduction in several crucial areas in the operating room [7, 8]. These three consistent steps have been instrumental in improving efficiency in the operating room and have allowed for the enhancement of quality and patient care for surgical patients. Given the vast number of similarities between the operating theater and endoscopy centers these same steps are also applicable to the field of GI.

## 3. Measuring Efficiency in Endoscopy Centers: Where Are We Now?

Along the same lines as lessons learned in anesthesia, in order to begin enhancing efficiency within an endoscopy center, one of the first steps is to clearly define the metrics that will be used to initially assess the organization and to eventually measure its success. A* metric* is any type of measurement used to gauge some quantifiable component of an organization's performance [37]. Moreover, once metrics are established and measured, comparing them against “the best practice” is the next step. A* benchmark* is a standard or point of reference in measuring or judging the current value or success of an organization in order to determine its success or overall performance in comparison to the performance of other similar organizations [38]. Metrics and benchmarks have been clearly defined in other aspects of healthcare such as the operating room; however, accepted and rigorously studied metrics/benchmarks are lacking for endoscopy centers. This void was highlighted by a conference of multiple endoscopy center directors in 2008 that determined there were either minimal or varying measurements employed that adequately assessed efficiency in the endoscopy center. Their recommendations were that there needed to be more “collect(ing) data in reference to personnel and unit management in endoscopy units” and “(promotion) of a set of standards for measuring efficiency in the endoscopy unit” needed to be pursued and more clearly elucidated [39, 40].

In an attempt to study efficiency within endoscopy centers, a few authors have established endoscopy center metrics ad hoc (Table 1). These metrics are modeled after ones developed by anesthesia where each aspect from the time when a patient arrives at the endoscopy center to the moment when they are discharged is dissected into its constituent parts and measured with respect to time. The most exhaustive research in the area of endoscopy center metrics has been performed by only a few groups [22, 24] who examined a number of time factors involved in patient flow through an endoscopy center. Consistently these groups examined preprocedure time, procedure time, room turnover time, and recovery time. However, efficiency measurements used in these studies, while being comprehensive and potentially useful in assessing overall flow and utilization of the endoscopy center, were labor intensive and cumbersome and were difficult to practically employ into most endoscopy centers. A more global approach was recently proposed whereby endoscopy center efficiency metrics were divided into three distinct categories: (1) structural, (2) process, and (3) outcome measures; unfortunately very little data has validated many of these proposed measures and again the practical implementation of them has been questioned [41]. In contrast to the anesthesia literature, no set of efficiency metrics has been adopted nor accepted.

While much research has focused on identifying and examining a laundry list of metrics, some studies have taken the approach of identifying and studying a few key metrics. For example, several studies have examined only one or two metrics such as procedure duration, recovery room time, and/or elapsed time between endoscopic procedures (e.g., turnover time of endoscopy room) in an effort to more easily study efficiency [30, 42–44]. While it is easier to measure one metric alone, none of these metrics have been accepted by gastrointestinal societies at the moment. Moreover, other metrics related to facility design, personnel, productivity, equipment, and patient satisfaction, which are widely used in other industries, are vaguer and not widely reported in the GI literature. Thus, while metrics are critical to measuring the efficiency of an endoscopy center, they have not been well studied nor reported in the literature, and more importantly which one(s) are the easiest and best measure of a center's efficiency is even less clear.

Once an endoscopy center has determined which set of metrics to utilize and measure, it is critical to have benchmarks. Initially, one may not need benchmarks as internal metrics can be used to gauge the success or failure of implementing process or technology changes. However, benchmarks serve to help one guide their organization in accordance with accepted industry standards and best practices and to further build their practice. Multiple industries such as the automobile, airline, and banking sectors have clearly defined benchmarks for which one measures success and operational/productivity improvements. Yet, as with metrics, few available and published benchmarks are known for endoscopy centers. Scant data is available for some measurements that are used in research, but again it is unclear whether these measurements represent the “optimal” assessment. Furthermore, no clear consensus has been reached on them. Additionally, of the few published benchmarks, there is considerable heterogeneity in their numbers with a multitude of confounding factors such as type of sedation utilized for each procedure, procedure type, and type of endoscopy center where the data was collected (outpatient ambulatory center versus tertiary hospital) (Table 2). Much of the remaining literature is less rigorous and focuses mostly on “expert opinion” with respect to suggested personnel, equipment, and facility requirements of endoscopy centers with no clear evidence to support such recommendations [34, 36] (Table 3). Consequently, there is minimal data on accepted metrics and benchmarks and available information is fraught with bias, inconsistencies, and lack of evidence. Thus, more robust and well-designed studies are needed to further determine a core set of optimal efficiency metrics as well as their associated benchmarks for endoscopy centers.

## 4. Examining Efficiency in Endoscopy Centers: An Observation and Modeling Approach

With an emphasis on cost containment and improving efficiency in healthcare two methods, (time and motion studies and discrete event simulation modeling), have been successfully advocated and performed in order to attain these goals. While using these two methods have been incredibly successful in a number of other healthcare areas, their use in endoscopy centers has been less evident. However, limited GI research has illustrated some promising results with using these two methods with respect to two areas examining several simultaneous changes to an endoscopy center versus studying one focused change.

With regard to the first area, two groups have employed time and motion studies and simulation modeling to fully examine a number of factors that impact endoscopy center efficiency [22, 24]. Initially, Harewood et al. in a prospective study at a large teaching hospital in Dublin, Ireland, utilized a “time and motion” approach to assess efficiency at their endoscopy center. After carefully measuring various timeframes they were able to calculate an “efficiency quotient” (e.g., proportion of time the endoscopist was engaged in performing the procedure or completing postprocedure paperwork) and then modelled various interrelated scenarios that could be implemented to increase overall efficiency [24]. More recently, Day and colleagues built upon this work by using time and motion studies and constructed a discrete event simulation model that evaluated multiple scenarios which were aimed at improving endoscopy center efficiency. Their work illustrated that weekly endoscopy center patterns were predictable and could provide insight into what potential changes were beneficial in a safety-net hospital endoscopy center. Moreover, they discovered that patient throughput as well as provider and nursing utilization was substantially increased with only simple changes such as realigning the endoscopy schedule with patient preferences and minimizing and streamlining the recovery room and preprocedure processes [22].

In the second area, smaller studies have modeled the effect on efficiency by examining one specific change to an endoscopy center rather than multiple, simultaneous ones. Some studies have focused on altering staffing specifically focusing on the endoscopist [30, 42, 44] and utilizing additional staff in the preprocedure process [24]. While such changes improve physician efficiency and utilization, they did so at a cost of impairing nonphysician staff utilization and suboptimizing facility utilization [30]. Using simulation modeling, others have discovered that identifying bottlenecks such as patient recovery [43, 45], reducing room turnover time [43, 44], modifying patient arrival schedule [42, 46], or reengineering patient scheduling [46, 47] improved efficiency and decreased patient stay. What is understood from all of these studies is the power that intense observation and simulation modeling can offer in better understanding the operations and how potential changes can affect the efficiency of an endoscopy center.

## 5. Optimizing GI Endoscopy Center Efficiency: Where to Begin?

**Figure 1:**
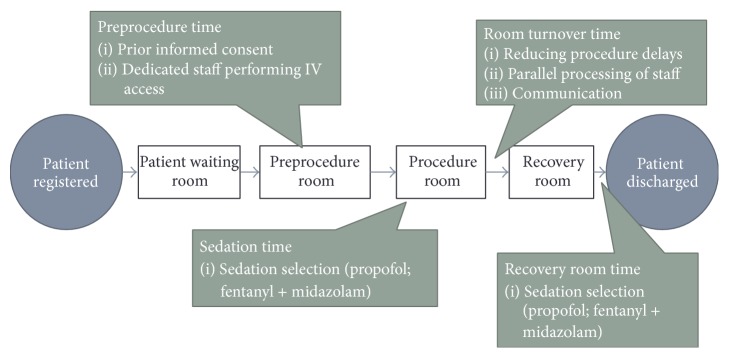
Factors that can streamline endoscopy center workflow processes and improve efficiency.

In assessing efficiency within endoscopy centers there are several potential areas where process improvements can be implemented. Process improvement methods have been demonstrated to remove inefficiencies within endoscopy centers. For example, Schembre and colleagues utilized manufacturing efficiency tools from the Toyota Corporation (e.g., LEAN methodology) to improve flow within their endoscopy center. After extensively studying patient and staff travel patterns, resource utilization, and equipment standardization, they then implemented several changes based on their observations. Consequently, they were successful in reducing patient waiting times while increasing procedure volume [48]. Through similar work a number of key areas have been identified for process improvement work in endoscopy centers; these areas include personnel utilization, patient scheduling, delays, room turnover time, and recovery room time. A framework for how to make improvement changes in each of these areas is discussed below (Figure 1).

### 5.1. Personnel Utilization

A crucial factor within an endoscopy center is the staff of it and how to best utilize staffing resources. Work has been conducted in this arena and can serve as a useful resource for endoscopy centers. With respect to nursing, some GI societies have proposed nursing staff models, but while being helpful it should be noted that no data exist to support or refute these recommendations [49]. Likewise, proposed models for utilizing endoscopists have been put forth. For example, A. Marasco and F. Marasco hypothesized that if facility space is a constraint in an endoscopy center, then it is more efficient to reduce room turnover time whereas if there is an abundance of endoscopy rooms, then it becomes more efficient to employ a “one-endoscopist-two-room” model [36]. This endoscopist model has been studied further; however, its results with respect to efficiency are inconclusive and conflicting. For example, using such a model has been shown in some studies to increase efficiency by 50% [24] and has been demonstrated to increase procedure volume by 11% [35], yet Rex using simulation modeling demonstrated that such an endoscopist model while increasing patients served and physician utilization, did so at a cost of the endoscopy center being suboptimized with patient length of stays increased and nonphysician staff utilization decreasing [24].

Another proposed endoscopist model is having nonphysicians (nurse practitioners/physician assistants) perform endoscopy. Numerous data have shown that nonphysicians can safely perform endoscopic procedures with similar quality to physicians [50–54]. Expanding the role of nonphysicians into performing endoscopy would allow endoscopy centers to increase services and access and allow gastroenterologists to focus their attention on more complex and demanding procedures/cases.

Lastly, how the entire team of providers is organized is a critical decision. Many advocate a “Pod layout” whereby a team is assigned to one physician/room for the day. This team is responsible for all aspects of patient care from preparation through recovery [34, 35]. This concept is modeled after the Japanese manufacturing sector whereby small groups work in a cohesive unit and such a model has proven to be both efficient and productive. However, how effective this model is compared with other proposed models has not been assessed. Thus, it is unclear which physician model to employ and even more so optimal ratios for other staff members within the endoscopy center have yet to be identified or studied. Crafting personnel models and determining which one best fits within a specific endoscopy center's practice would undoubtedly improve efficiency.

### 5.2. Patient Scheduling

Within ambulatory surgery centers several studies have demonstrated that shortening procedure time does not improve efficiency, but rather other factors such as scheduling and operational improvements are more instrumental. One proposed method for scheduling procedures focuses on utilizing open access endoscopy (e.g., scheduling and performing endoscopic procedures without a formal GI consultation/office visit). Such a method can improve patient access to both clinic and endoscopy, but some limitations may exist. For example, some studies suggest that open access yields more inappropriate indications for procedures with a reported 10–28% prevalence of incorrectly scheduled procedures [34]. A second approach to scheduling patients is the utilization of block time for providers and has been recommended as the most efficient use of creating an endoscopy schedule [55]; however, data for this recommendation only hails from the anesthesia literature [56, 57]. Additionally, mathematical models have been developed to help improve use rate, utilize waiting time characteristics, and incorporate overbooking into endoscopy centers, but implementation of these models into endoscopy center practice has not been reported [58]. While it is not clear which method would improve efficiency, what is evident from the literature is that through observation of workflow and understanding endoscopy center patterns one can better align the endoscopy center schedule to better improve throughput and staff and facility utilization [22].

### 5.3. Procedure Delays

Equally important to scheduling factors is minimizing delays within the endoscopy center. There are two types of endoscopy center delays: patient and procedure related. There are a number of interventions the endoscopy center can employ to minimize patient related delays. These include ensuring that patient instructions are clear, providing excellent directions to the endoscopy center, performing an advance call to patients to review preparatory instructions/medications, and ensuring an efficient check-in process once the patient arrives at the endoscopy center. Along these lines, a number of modalities have been shown to improve patient adherence to pre-endoscopy instructions and reduce patient related delays; such modalities include multimedia, interactive computer programs, and education classes [59]. On the other hand, procedure related delays are similarly important to minimize. In this area physicians are overwhelmingly responsible for such delays [60]. These delays have a significant impact on workflow processes, add to patient waiting time, and increase room turnover time. Physician related delays are usually the result of multitasking and/or performing other tasks not related to endoscopy or in some cases endoscopists may exceed their scheduled procedure times when performing endoscopy. It is crucial to address these issues with providers, monitor and share this data with physicians, and have mechanisms in place to deal with physician behavior.

### 5.4. Sedation

While reducing endoscopic procedure time does not appear to increase efficiency of an endoscopy center [30], other factors that occur within the procedure room can improve efficiency. In particular, induction/sedation time can be reduced based on the type of sedation utilized leading to less overall time in a procedure room and thus greater efficiency. Significant attention has been focused on the use of propofol which has clearly demonstrated benefits on endoscopy center efficiency in a number of areas, one of which is reducing overall sedation time [28]. However, controversy surrounds the administration of propofol by endoscopists/nurses and the addition of an anesthesiologist to the care team to provide such a service, while increasing overall efficiency, does so at a dramatic financial cost. Alternatives to propofol may include changing the type of moderate sedation used. Recently, the use of combination midazolam/fentanyl was shown to reduce total procedure time (due to shorter induction-to-intubation time) for patients undergoing upper endoscopies with overall efficiency rising by 22% compared with midazolam/meperidine use for the same procedure [25]. Clearly, the sedation type administered should be examined as it has an impact on several components of endoscopy center efficiency.

### 5.5. Room Turnover Time

One area that has been used as a marker for improving efficiency is reducing endoscopy room turnover time. Using procedure volume per hour as a marker for efficiency, Zamir and Rex demonstrated a clear inverse relationship between this marker and room turnover time [44]. Yet, the majority of tasks associated with room turnover time are fixed and can be difficult to streamline. However, previous work in the operating room has realized this challenge and some work has demonstrated that parallel processing of tasks among staff members (e.g., simultaneously performing several patient related tasks at the same time) can lead to a dramatic reduction in operating room turnover time [7, 8]. Additionally clear and immediate communication of when a procedure is completed (in order to begin the room turnover process) and clearly identifying staff roles in this process are critical elements at minimizing room turnover time.

### 5.6. Recovery Room

Lastly, reducing recovery room time can help increase efficiency. With regard to recovery room time, Grossman et al. modeled an ambulatory surgery center and demonstrated that recovery time was the main bottleneck. In fact, a 50% reduction in recovery time increased the number of patients per room per day and shortened the overall length of stay of patients [43]. However, how to address this bottleneck has been less well studied. Aside from increasing the physical space of the recovery room the only specific intervention proposed to reduce this time has been sedation related. The use of propofol in some centers [28], using one sedating medication compared with two medications [26], and using midazolam/fentanyl for moderate sedation [25] all reduce recovery room time and increase overall procedure volume in endoscopy centers.

## 6. Conclusion

Improving efficiency in endoscopy centers has been an increasingly important topic. Overall, there are three key steps to consider when beginning the journey of improving endoscopy center efficiency. The first step in improving efficiency within an endoscopy center is to determine which metrics would be the most relevant and feasible to measure within one's organization. At the same time, it is crucial to remember that metrics help identify where to improve but do not directly cause improvements to take place. Also, while no accepted or standardized benchmarks have been adopted for endoscopy centers, there is some limited data available for which one can compare their data measurements. Secondly, an initial assessment of potential areas where inefficiencies may exist in an endoscopy center should be conducted with particular focuses on patient flow, staffing, facility, and equipment. Along these lines, there is evidence to suggest that time and motion studies coupled with simulation modeling can aid endoscopy centers in better understanding their operations and how potential changes can affect the efficiency of their center. Finally, a number of areas exist where endoscopy centers can focus in order to begin improvement work in efficiency. Examining endoscopist models, minimizing patient and procedure related delays, utilizing block scheduling, reducing room turnover time through clear communication and role definition, and considering the types of sedation administered are factors that have been demonstrated to impact efficiency and procedure volume in endoscopy centers. Lastly, one has to tailor their proposed innovations to be in alignment with the goals of their organization and patient population.

## Figures and Tables

**Table 1:** Proposed endoscopy center efficiency metrics.

Operational metrics	Waiting room time (time between patient check-in and transport to preprocedure room)
	preprocedure room time (time between patient entering preprocedure room and transport to procedure room) (i) Preprocedure nursing time (placing IV, completing paperwork, vitals, and assessment) (ii) Preprocedure physician time (history and physical, obtaining consent, and paperwork)
	Procedure room time (time between patient entering procedure room and patient transported out of procedure room) (i) Procedure duration (time of insertion of endoscope to its removal) (ii) Procedure room nursing time (paperwork) (iii) Physician postprocedure time (paperwork, generating endoscopy report)
	Sedation time (time of initial dose of sedating medication to beginning of procedure)
	Recovery room time (time between patient returning to recovery room and discharged from endoscopy center) (i) Patient recovery time (the time when patient arrives in recovery room to receiving discharge instructions) (ii) Discharge instruction time (the time when patient receives discharge instructions to when patient leaves the recovery room)
	Procedure room turnover time (the time when patient is transported to recovery room and procedure room is ready to accept the next patient)
	Total duration of patient in endoscopy center (the time when patient checks in and when patient is discharged)
	Total facility time (time when first patient checks-in to when last patient is discharged)

Productivity metrics	Number of procedures/hour
	Number of patients/physician/day
	Number of patients/procedure room/day
	Procedure volume (i) Annual procedure volume = number of endoscopic procedures performed in 1 year (ii) Procedure waiting time = mean waiting time for all patients undergoing an endoscopic procedure in 1 year (in weeks) (iii) Annual procedure demand = (annual procedure volume) + (annual procedure volume × (procedure waiting time/52))
	Number of cancellations/day (or month)
	Physician utilization (proportion of the time when physician is engaged in procedure and completing procedure related paperwork)
	Nursing utilization (proportion of the time when nurse is engaged in procedure and completing procedure related paperwork)

Personnel/staff metrics	Number of physicians assigned to an endoscopy room/center
	Number of nurses assigned to an endoscopy room/center
	Number of nurses assigned to a preprocedure room/recovery room
	Number of endoscope reprocessor(s) assigned to an endoscopy center
	Number of ancillary staff assigned to an endoscopy center

Facility metrics	Size of endoscopy room
	Number of endoscope reprocessing rooms
	Number of beds/preprocedure room/endoscopy room
	Number of beds/recovery room/endoscopy room

Equipment metrics	Number of colonoscopes/endoscopy room
	Number of upper endoscopes/endoscopy room
	Number of pieces of advanced endoscopic equipment/endoscopy center
	Time required to reprocess 1 endoscope
	Number of automatic reprocessing machines/endoscopy room

Patient satisfaction metrics	Length of time from scheduling of endoscopic procedure to day of procedure
	On-time start of performing an endoscopic procedure
	Number of patient no-shows/day (or month)
	Patient satisfaction surveys (i.e., Press Ganey)

**Table 2:** Reported endoscopy center benchmarks based on reported literature.

Operational benchmarks	Esophagogastroduodenoscopy (EGD)	Colonoscopy
On-time procedure start (%) [22, 23]	53.3–75.0	55.0–75.0
Preprocedure time (min) [22, 24]	6.0–20.9	3.0–22.3
Procedure duration (min) [22, 24–26]	3.0–31.1	14.0–42.0
Sedation time (min)		
Moderate sedation^∗^ [22, 24–27]	5.0–10.0	2.1–11.2
Propofol [27–29]	2.1–3.6	2.1
Room turnover time (min) [22, 24, 26, 30]	3.0–26.6	2.0–26.6
Recovery room time (min)		
Moderate sedation^∗^ [22, 24, 26, 27]	9.1–50.2	14.0–61.0
Propofol [26–28, 31, 32]	3.4–15.0	14.3–18.0
Endoscopist completing paperwork after procedure (min) [24]	2.0	3.0

^∗^Moderate sedation includes midazolam/fentanyl, midazolam/meperidine, and opioid alone.

**Table 3:** Reported endoscopy center benchmarks based on expert opinion.

Productivity benchmarks	
Number of procedures/room/day [33]	14–16
Number of patients/room/half-day	6

Personnel/staff benchmarks	
Number of physicians/room	1
Number of nurses/room [34]	1.5–2
Number of reprocessors per center	1-2

Equipment benchmarks	
Number of endoscopes: endoscopy room	2 upper endoscopes and 2 colonoscopes: procedure room
Mean time of reprocessing endoscopes (min)	30
Number of automatic endoscope reprocessors: procedure rooms [35]	1.5–2: 1

Facility benchmarks	
Size of endoscopy room [34]	220/300 square feet
Number of recovery beds: procedure room [35, 36]	2-3: 1
Number of preprocedure beds: procedure room [35, 36]	2: 1
